# Comparing position sense and isokinetic strength of the muscles of elbow joint between aikidokas and non-athlete people

**DOI:** 10.1186/s13102-023-00677-5

**Published:** 2023-06-13

**Authors:** Rezvan Mirsalari, Amin Kordi Yoosefinejad, Farzaneh Yazdani, Farzaneh Haghighat, Ali Asghar Amiripanah, Saeid Parandavarfard

**Affiliations:** 1 Student Research Committee, Shiraz University of Medical Sciences, Shiraz, Iran; 2 Physical Therapy Department, School of Rehabilitation Sciences, Shiraz University of Medical Sciences, Shiraz, Iran; 3 Rehabilitation Sciences Research Center, Shiraz University of Medical Sciences, Shiraz, Iran; 4 Jiyushinkai Aikido director, Head of central (Takhti) Dojo, Shiraz, Iran; 5 Head of Technical Committee, Jiyushinkai Aikido director, Head of Fajr Dojo, Shiraz, Iran

**Keywords:** Aikido, Joint position sense, Isokinetic, Strength, Elbow

## Abstract

**Background:**

Aikido is a martial art comprises of locking techniques and falls. During the locking techniques, the elbow joint is forced into extended position. Moreover, the elbow hits the ground during the falling techniques. These might compromise joint position sense (JPS). The objectives of this study were to compare JPS and strength of the muscles of elbow joint between Aikidokas and a non-athlete group and to evaluate the correlation between JPS and muscle strength among Aikidokas.

**Methods:**

All male Jiyushinkai style Aikidokas and a healthy matched non-athlete group participated in this cross-sectional study. Passive JPS at a speed of 4°/s and the isokinetic strength of elbow flexors and extensors were assessed.

**Results:**

Evaluating the isokinetic parameters revealed no significantly difference between the groups in either flexion or extension at speeds of 60 (P-value range: 0.2–0.99) and 120 °/s (P-value range: 0.05–0.96). Also, the groups had no significant difference regarding different types of reconstruction error including constant error (P-value range: 0.38–0.91), variable error (P-value range: 0.09–0.87), and total variability (P-value range: 0.30–0.80). Moreover, very weak to weak correlation was observed between isokinetic parameters and passive JPS (*r*-value range: 0.01–0.39).

**Conclusions:**

JPS was not impaired in Aikidokas in spite of the repetitive stress applied to the elbow joint during the performance of Aikido techniques. The lack of significant difference in isokinetic between Aikidokas and healthy non-athletes, and the absence of an acceptable correlation between IPS and muscle strength in Aikidokas, might be attributed to the soft nature of Aikido.

## Introduction

Aikido is a martial art with a defensive nature that people of variety of ages and performance levels can take part in [1]. Ikkyo and sankyo are two locking technics commonly used in Aikido. Ikkyo simultaneously affects joint and nerve biomechanics. During the performance of the technique, the elbow joint is forced into an extended position, while pressure is applied to the nerve on the medial side of the arm [2, 3]. Sankyo is another locking technique used by intermediate to advanced Aikidokas to control the attacker with minimal effort and maximal efficiency [2, 3]. Since performed bilaterally, the locking techniques predispose elbow joint of dominant and non-dominant upper limbs to injury. In fallings (Okemi) techniques, the body hits the ground in an arc commencing with the elbows and continuing on the shoulder and hip joints [4]. Therefore, the elbow joint is vulnerable to injury during the Okemi techniques as well. According to Diesselhorst et al., 19% of the injuries in martial arts were related to the elbow [5]. Moreover, Zetaruk et al. compared five different martial arts and found that the most injury occurrences (28%) was noted in Aikidokas [6]. Furthermore, 27% of Aikido-related injuries involve upper limb joints [7].

The term “proprioception” was first introduced by Sherrington in 1906, who described it as a form of feedback from the limb to central nervous system (CNS) [8]. Proprioception is the product of sensory information provided by nerve terminals known as mechanoreceptors. Mechanoreceptors are located within muscles, ligaments, joints, and fascia. Muscle spindles are considered the most important source of proprioception being highly sensitive to movement and having different distributions throughout the body, reflecting different functional requirements [9]. Repetitive stress to the muscle spindles of the muscles of the elbow joint may compel them to send inappropriate afferent signals to the CNS, which may result in impaired proprioception. Problems with joint capsule, tendons, ligaments and muscles are regarded the main cause of elbow complaints leading to proprioception deficit [10]. As mentioned above, Aikido techniques affect the tendons and other structures surrounding the elbow joint. Proprioception deficits can play a role in several tendon diseases like that proven by Juul-Kristensen et al. for patients with lateral epicondylitis [11]. Despite its great importance, evaluation of elbow proprioception, particularly in martial arts like Aikido is still in its infancy. Ozden and colleagues found that more errors seem to occur in elbow proprioception in comparison to shoulder proprioception during sport activities [12].

Muscular strength is one of the most important factors plausibly affecting human performance, enabling athletes to overcome the external loads applied to the body and enabling motion. Muscles traversing or originating from the elbow, provide the stability of the joint in all the movement planes [13]. Muscle strength and proprioception are combined together in functional motor performance in ADLs and sport.

Considering the vital role of joint proprioception in various aspects of sensorimotor control, its thorough evaluation is particularly important in people who are susceptible to proprioceptive injuries [14]. Regarding the basic role of muscle spindles in encoding proprioceptive and force-related variables, we hypothesized that there might be a strong correlation between the changes in proprioception and muscle strength among Aikidokas [15]. Also, regarding the corroborating role of muscle strength and proprioception, it could be speculated that repetitive strain to elbow joint during practicing Aikido, might damage the mechanoreceptors, resulting in deafferentation and subsequent decrease in elbow proprioception. Accordingly, following damage to mechanoreceptors, the neuromuscular response required for joint stability would be disrupted. Hence, the stability deteriorates [12]. However, there is a dearth of studies evaluating the possibility of this relationship. Hence, the objectives of this study were: (1) to compare joint position sense and strength of the muscles of elbow joint between Aikidokas and healthy matched people and (2) to see if there were considerable correlation between joint position sense and muscle strength among Aikidokas.

## Methods

### Experimental approach

It was a cross-sectional study conducted between January and May 2022 at the Rehabilitation Research Center of Shiraz University of Medical Sciences. The study was approved by the Ethics Committee of the Vice Chancellor of Research, Shiraz University of Medical Sciences in accordance with the standards of the Helsinki declaration (Ethics code: IR.SUMS.REHAB.REC.1399.040). All the participants signed an informed consent form after being given a detailed explanation about the study procedure.

The inclusion criteria for the Aikido group were as follows: (1) at least one-year experience in practicing Aikido (2) practice 2–4 days a week for 1.5 to 2 h [16].

Individuals with diseases affecting musculoskeletal or neurological systems and those who had participated in other sports [17] were excluded from the study.

### Participants

The participants were male Aikidokas who had accomplished first or second kyu (intermediate) degrees and all dan (advanced) degrees [16]. Our population consisted of all Jiyushinkai style Aikidokas of Shiraz practicing in Fajr and Takhti dojos. Twenty-one male Aikidokas participated in our study. Both dominant and non-dominant upper limbs were evaluated. The control group had no history of pain or injury in the elbow area in the last 6 months and were matched with Aikidokas in terms of age and body mass index (BMI) [18].

### Procedures

To identify the dominant limb, Edinburgh Dominance Hand Questionnaire was used. The questionnaire was designed by Oldfield in 1971 to determine hand preference in normal population [19]. This questionnaire includes questions on performing daily activities.

Passive joint position sense and isokinetic strength of elbow flexors and extensors were evaluated using the Biodex system 4 (Biodex Medical Systems, Inc, Shirley, New York) with acceptable reliability and repeatability to investigate athletic performance [20]. Participants used blindfolds and headphones during the sense of position test [14, 18]. To measure the sense of position, the hip and knee joints were bent to approximately 90 degrees and the trunk was fixed with a strap. The forearm was placed in a neutral position on the armrest [16, 21]. The axis of the dynamometer was aligned with the axis of the elbow joint [21, 22]. To compensate for the effect of gravity, the hand was comfortably placed on a lever arm and the weight of the hand was measured and removed.

#### Position sense assessment

Passive joint position sense was performed at a speed of 4°/s [1]. Before the main test, the participants had a familiarization session with tests. The starting angle of the movement was at 60 degrees of flexion. The target angle was randomly selected at either 30 or 90 degrees of flexion. During flexion test, the elbow was shifted toward the target angle (90-degree flexion) passively, and in order for the person to learn the position of the hand, the device was stopped at the target angle for five seconds. The target angle was recorded in the device. Then, the forearm was returned to the starting angle (60 degrees of flexion). When the elbow reached the target angle by the device, the participant pressed the stop button and the error value was recorded. Following a 60-second rest, the next step commenced. The elbow was moved to the other target angle (30-degree flexion) by the device, and the above steps were repeated. Each trial repeated 3 times [23], and the absolute error of each trial was recorded for future analysis. To have a precise measurement and better understanding of the possible mechanisms involved in passive joint position reconstruction, we calculated other measures of error including constant error (CE), variable error (VE), and total variability (E) [24]. CE is calculated using the following formula:

CE = Σ (x_i_ − T) / n.

where x_i_ is the score on trial i, “T” is the target, and “n” is the number of trials the participant performed. CE is given in units representing the amount and direction of deviation relative to the target, sometimes called bias.

VE is a measure of inconsistency in movement outcome and is calculated by the formula:

VE= $$\surd {\Sigma }($$x_i_ – M_)_^2^ / n.

where “M” is the participant’s average movement, measured in the same units as the scores for the trial [24].

“E” could be thought of as the measure of overall error. It represents the overall measure of how successful the participant was in achieving the target. Total variability is calculated by the following formula:

E= $$\surd {\Sigma }($$x_i_ − T)^2^ /n [24].

#### Isokinetic strength assessment

The dynamometer was calibrated according to the manufacturer’s instructions. The position of the participants during isokinetic tests was the same as joint position sense. Subjects performed concentric and eccentric contractions as warm-up at two speeds (60 and 120°/s) before performing the test [25]. The starting angle of the movement was at 0 degrees of flexion. The participant was asked to perform concentric contractions from 0° to 90° flexion with an angular speed of 60°/s and 120°/s. Eccentric contractions from 90° flexion to 0° flexion with a speed of -60°/s and − 120°/s were performed as well. The participants were instructed to perform the test with maximal effort. Each trial repeated five times at a speed of 60 and 120 °/s, with a 60-second rest between the trials [26].

The evaluated parameters were peak torque normalized to body weight, maximum repetition total work, total work, and average power.

### Statistical analysis

Data were analyzed using SPSS statistical software (IBM SPSS Statistics for Windows, Version 25.0. Armonk, NY: IBM Corp). The normal distribution of data was verified by Shapiro-Wilk test. Descriptive data are presented as mean ± standard deviation. Between-group comparison was performed using univariate ANOVA. The effect size was evaluated using partial eta square. The partial eta square values are interpreted as 0.01 equal to small effects, 0.06 as moderate effect, and 0.14 as a large effect. The correlation between JPS and isokinetic parameters was evaluated by Pearson correlation coefficient (*r*). The “*r*” values were interpreted as: 0–0.19 = very weak correlation, 0.2–0.39 = weak correlation, 0.4–0.69 = moderate correlation, 0.7–0.89 = strong correlation, and 0.9–1.00 = very strong correlation. P-value < 0.05 was considered statistically significant [27].

## Results

Demographic data of the participants is summarized in Table 1.No significant difference was observed between the groups regarding demographic data.

**Table 1:** Demographic data of the participants

variable	Aikido group (n = 25) mean ± SD*	Control group (n = 25) mean ± SD	p-value
Age (years)	35.84 ± 10.19	30.80 ± 7.82	0.05
BMI † (kg/m2)	27.05 ± 4.16	26.49 ± 3.33	0.60
Dominant hand (Rt/Lt) ‡	23/2	24/1	----
Aikido experience (years)	9.82 ± 6.40	----	----

*: Standard Deviation, †: Body Mass Index, ‡: Right/Left

Comparison of isokinetic data between the groups at speeds of 60˚/sec and 120˚/sec are depicted in Tables 2 and 3 respectively. The isokinetic parameters were not significantly different between the groups in either direction at both speeds.

**Table 2:** Comparison of isokinetic data between the groups at a speed of 60˚/sec in flexion and extension

variable	Rt/Lt†	Flexion/ Extension	Aikido group (n = 25) mean ± SD £ (CI ¥ 95% (lowerlimit, upper limit))	Control group (n = 25) mean ± SD (CI 95% (lower limit, upper limit))	df *	Mean Square	F-value	η_p_^2^ ‽	p-value
PT/BW‡ (%)	Rt	Extension	16.96 ± 3.86 (15.37,18.56)	16.50 ± 4.98 (14.45,18.56)	1, 48	2.60	0.13	0.003	0.72
		Flexion	19.34 ± 3.58 (17.86,20.82)	20.48 ± 3.79 (18.92,22.05)	1, 48	16.36	1.20	0.024	0.29
	Lt	Extension	16.71 ± 3.55 (15.24,18.17)	16.38 ± 4.07 (14.70,18.06)	1, 48	1.31	0.09	0.002	0.77
		Flexion	19.26 ± 3.34 (17.88,20.63)	19.91 ± 3.57 (18.43,21.38)	1, 48	5.31	0.45	0.009	0.51
Max Rep Total work (Nm)	Rt	Extension	37.40 ± 10.68 (32.99,41.81)	37.44 ± 10.94 (32.93,41.96)	1, 48	0.024	> 0.001	> 0.001	0.99
		Flexion	45.80 ± 9.08 (42.05,49.55)	49.12 ± 8.92 (45.44,52.81)	1, 48	138.11	1.70	0.034	0.20
	Lt	Extension	38.17 ± 8.86 (34.52,41.83)	37.03 ± 9.83 (32.98,41.09)	1, 48	16.25	0.19	0.004	0.67
		Flexion	46.24 ± 9.30 (42.40,50.07)	47.85 ± 8.05 (44.53,51.17)	1, 48	32.81	0.43	0.009	0.51
Total work (Nm)	Rt	Extension	166.24 ± 53.08 (193.44,228.17)	163.69 ± 56.05 (140.55,186.83)	1, 48	53.08	0.03	0.001	0.87
		Flexion	210.84 ± 42.07 (193.44,228.17)	226.78 ± 44.60 (208.40,245.19)	1, 48	3190.41	1.70	0.034	0.20
	Lt	Extension	168.43 ± 43.20 (150.60,186.26)	161.60 ± 48.96 (141.39,181.80)	1, 48	583.45	0.27	0.006	0.60
		Flexion	214.21 ± 45.16 (195.56,232.85)	221.37 ± 42.11 (203.98,238.75)	1, 48	640.82	0.34	0.007	0.56
Average power (watt)	Rt	Extension	28.34 ± 8.97 (24.63,32.04)	27.68 ± 10.21 (23.46,31.89)	1, 48	5.45	0.06	0.001	0.81
		Flexion	34.61 ± 6.83 (31.79,37.43)	36.41 ± 8.80 (32.77,40.04)	1, 48	40.50	0.65	0.013	0.42
	Lt	Extension	28.43 ± 7.50 (25.34,31.53)	26.53 ± 8.07 (23.19,29.86)	1, 48	45.32	0.75	0.015	0.39
		Flexion	34.73 ± 7.90 (31.47,37.99)	35.55 ± 7.21 (32.57,38.53)	1, 48	8.41	0.15	0.003	0.70

†: Right/ Left, ‡: Peak Torque normalized to Body Weight, £: Standard Deviation, ¥: Confidence Interval, *: Degrees of Freedom, ‽: Partial Eta Square

**Table 3:** Comparison of isokinetic data between the groups at a speed of 120˚/sec in flexion and extension

variable	Rt/Lt†	Flexion/Extension	Aikido group (n = 25) mean ± SD £ (CI ¥ 95% (lower limit, upper limit))	Control group (n = 25) mean ± SD (CI 95% (lower limit, upper limit))	df *	Mean Square	F-value	η_p_^2^ ‽	p-value
PT/BW ‡	Rt	Extension	14.93 ± 3.26 (13.58,16.27)	13.41 ± 4.02 (11.75,15.07)	1, 48	28.88	2.15	0.04	0.15
		Flexion	15.69 ± 3.32 (14.32,17.06)	16.33 ± 2.83 (15.17,17.50)	1, 48	5.18	0.54	0.01	0.46
	Lt	Extension	14.68 ± 3.88 (13.28,16.08)	12.88 ± 3.71 (11.35,14.41)	1, 48	40.32	3.20	0.05	0.08
		Flexion	15.90 ± 2.92 (14.70,17.09)	15.68 ± 3.13 (13.39,16.97)	1, 48	0.63	0.06	0.01	0.79
Max Rep Total work (Nm)	Rt	Extension	32.27 ± 9.53 (28.33,36.20)	29.75 ± 9.44 (25.85,33.64)	1, 48	79.38	0.88	0.02	0.35
		Flexion	38.10 ± 7.41 (35.04,41.16)	38.93 ± 7.75 (35.73,42.13)	1, 48	8.65	0.15	0.003	0.70
	Lt	Extension	32.36 ± 10.35 (28.09,36.64)	28.43 ± 10.13 (24.24,32.61)	1, 48	193.65	1.85	0.04	0.18
		Flexion	38.39 ± 7.73 (35.19,41.58)	38.17 ± 9.61 (34.20,42.14)	1, 48	0.58	0.008	> 0.001	0.93
Total work (Nm)	Rt	Extension	142.49 ± 46.66 (123.23,161.75)	127.44 ± 49.95 (106.82,148.06)	1, 48	2829.02	1.21	0.02	0.27
		Flexion	178.00 ± 37.25 (162.62,193.37)	179.26 ± 37.73 (163.69,194.84)	1, 48	20.10	0.01	> 0.001	0.90
	Lt	Extension	147.31 ± 50.60 (126.27,168.35)	121.63 ± 51.61 (100.33,142.93)	1, 48	8234.28	3.13	0.006	0.08
		Flexion	178.63 ± 37.02 (163.35,193.91)	173.14 ± 44.13 (154.92,191.35)	1, 48	377.03	0.64	0.005	0.64
Average power (watt)	Rt	Extension	45.01 ± 15.19 (38.74,51.28)	39.20 ± 16.63 (32.33,46.06)	1, 48	422.24	0.20	0.03	0.20
		Flexion	52.18 ± 11.08 (47.60,56.75)	52.01 ± 12.54 (46.83,57.19)	1 48	0.34	0.002	> 0.001	0.96
	Lt	Extension	46.50 ± 15.76 (39.09,52.10)	36.78 ± 16.21 (30.09,43.47)	1, 48	971.52	3.80	0.06	0.05
		Flexion	52.44 ± 11.94 (47.50,57.36)	49.70 ± 13.88 (43.97,55.43)	1, 48	93.30	0.56	0.01	0.46

†: Right/ Left, ‡: Peak Torque normalized to Body Weight, £: Standard Deviation, ¥: Confidence Interval,, *: Degrees of Freedom, ‽: Partial Eta Square

The between-group comparison of constant error, variable error, and total variability during passive reconstruction of target angles (30 and 90 degrees) are depicted in Table 4.

**Table 4:** Comparison of different errors during passive reconstruction of target angles between the groups

variable	Rt/Lt	Reconstruction angles	Aikido group (n = 25)	Control group (n = 25)	CI 95% of difference (lower limit, upper limit)	df *	Mean Square	F-value	η_p_^2^ ‽	p-value
CE* (degrees)	Rt	30	2.14 ± 3.82	2.27 ± 3.96	-2.33, 2.09	1, 48	0.21	0.01	0.04	0.91
		90	-1.32 ± 4.18	-0.98 ± 4.08	-2.70,2.00	1, 48	1.45	0.08	> 0.01	0.77
	Lt	30	2.52 ± 3.06	3.38 ± 3.88	-2.85,1.12	1, 48	9.25	0.76	0.01	0.38
		90	-0.97 ± 4.03	-1.52 ± 3.77	-1.67,2.77	1, 48	3.78	0.25	> 0.01	0.62
VE† (degrees)	Rt	30	2.25 ± 1.42	2.32 ± 1.50	-0.90,0.76	1, 48	0.06	0.03	> 0.01	0.87
		90	2.86 ± 1.46	2.21 ± 1.45	-0.75,0.90	1, 48	5.28	2.49	0.05	0.86
	Lt	30	1.88 ± 1.29	2.58 ± 1.59	-1.52,0.12	1, 48	6.13	2.92	0.05	0.09
		90	2.17 ± 1.18	2.73 ± 1.92	-1.46, 0.35	1, 48	3.92	1.54	0.03	0.21
E‡(degrees)	Rt	30	3.46 ± 2.62	3.96 ± 2.16	-1.86,0.86	1, 48	3.13	0.54	0.01	0.46
		90	3.27 ± 2.84	3.46 ± 2.28	-1.65,1.28	1, 48	0.45	0.07	> 0.01	0.80
	Lt	30	3.27 ± 2.19	4.07 ± 3.12	-2.34,0.74	1, 48	8.00	1.10	0.02	0.30
		90	3.28 ± 2.46	3.10 ± 2.58	-1.25,1.61	1, 48	0.41	0.06	> 0.01	0.80

*: Constant error, †: Variable error, ‡: Total variability

The groups had no significant difference regarding different types of reconstruction error.

Regarding the correlation between the isokinetic strength at speeds of 60˚/sec and 120˚/sec, and JPS at both 30- and 90-degrees target angles, “very weak” to “weak” correlations were observed (Table 5).

**Table 5:** Correlation between the isokinetic strength at speeds of 60˚/sec and 120˚/sec, and JPS at both 30- and 90-degrees target angles

variable	Flexion/Extension	Speed (°/s)	Rt/Lt	JPS † (30°)	JPS (90°)
PT/BW ‡	Flexion	60	Lt	0.02 £	-0.02
			Rt	0.16	-0.26
		120	Lt	0.12	-0.16
			Rt	0.09	-0.19
	Extension	60	Lt	-0.07	-0.08
			Rt	0.25	-0.31
		120	Lt	-0.06	-0.09
			Rt	0.26	-0.37
Maximum work (Nm)	Flexion	60	Lt	0.03	-0.06
			Rt	-0.08	-0.31
		120	Lt	0.05	-0.14
			Rt	0.04	-0.25
	Extension	60	Lt	-0.11	-0.23
			Rt	0.21	-0.31
		120	Lt	0.11	-0.24
			Rt	0.29	-0.39
Total work (Nm)	Flexion	60	Lt	-0.03	-0.12
			Rt	-0.02	-0.29
		120	Lt	0.05	-0.07
			Rt	-0.35	0.08
	Extension	60	Lt	0.02	-0.14
			Rt	0.23	-0.33
		120	Lt	0.06	-0.22
			Rt	0.28	-0.37
Average power (watt)	Flexion	60	Lt	-0.03	-0.13
			Rt	0.08	-0.31
		120	Lt	-0.06	-0.07
			Rt	0.05	-0.21
	Extension	60	Lt	0.10	-0.24
			Rt	0.24	-0.36
		120	Lt	0.05	-0.21
			Rt	-0.37	0.01

‡: Peak Torque to Body Weight, †: Joint Position Sense, £: Pearson’s Correlation Coefficient

## Discussion

This study was two-fold: First, to compare the elbow JPS and isokinetic strength of elbow joint muscles between asymptomatic Aikidokas and matched healthy non-athletes; and second, to evaluate the relationship between the elbow JPS and muscle strength in Aikidokas.

The results showed that the elbow JPS and the isokinetic strength of the elbow flexors and extensors did not differ significantly between the groups, and no considerable correlation was found between the elbow JPS and the isokinetic strength of the elbow muscles in Aikidokas.

The lack of evidence evaluating elbow joint proprioception and muscle strength and investigating the probable correlation between them in Aikidokas, preclude us to compare the results of the present study with those of the previous ones. A previous study by Sanati et al., [1] also found no significant difference in wrist JPS between the male Aikidokas and healthy non-athlete matched group. Our results indicated that although the elbow joint is frequently encountered to potentially deleterious forces, including repeated twisting and locking maneuvers in Aikidokas, the elbow JPS was not deteriorated. Although this could be promising, some points should not be ignored. According to Firat and Uysal [23], the proprioceptive ability of the elbow joint, as an intermediate joint, is not merely depend upon its structures, but it also supplied by the wrist and shoulder elements. Therefore, it has been suggested that the evaluation and treatment of elbow joint proprioception should be designed in a complementary approach and should not solely focus on the elbow joint. In contrast to our findings, Niespodzinski et al. found that untrained boys had higher errors in elbow JPS than young and adult gymnasts [28]. This controversy could be attributed to the fact that gymnastic training peculiarly consisted of explosive and strength endurance exercises with body weight load, as well as coordination training [28]. Cho and Park found a significant correlation between JPS and peroneal strength in patients with chronic lateral ankle instability [29]. It should be noted that their participants suffered from chronic lateral ankle instability, while ours had no apparent disability.

Considering the Aikido as a martial art, rather than a competitive martial sport, it makes sense that there is no difference in the isokinetic strength of the elbow flexor and extensor muscles between the Aikidokas and healthy matched people. A previous study by Fong et al. [30] found that training in Ving Tsun martial art, could improve elbow extensor isometric peak force in middle-aged and older adults. Ving Tsun is characterized by rapid and forceful punching and arm techniques and is more strenuous than Aikido; thus, it may be a suitable exercise for practitioners to improve their muscle strength.

No correlation was found between the muscle strength and JPS at the elbow joint in Aikidokas. There is a lack of literature on the association between muscle strength and proprioception. strength training has been demonstrated to improve JPS at the shoulder [31] and ankle joint [32] in asymptomatic individuals and those with functionally unstable ankles, respectively. As mentioned earlier, Aikido does not directly focus on the strength training programs; thus, this may explain the lack of correlation between muscle strength and JPS of the Aikidokas. In line with our findings, Sanati et al. [1] reported no moderate to strong correlation between wrist JPS and muscle strength either.

This was the first study to compare the elbow JPS and muscles strength between Aikidokas and matched healthy non-athletes and also, the first one to evaluate the correlation between elbow JPS and muscle strength in Aikidokas. This study had some limitations. First, it was conducted only on male Aikidokas; so, the results could not be generalized to female Aikidokas. Second, we recruited Aikidokas of Jiyushinkai style; therefore, the results cannot be generalized to Aikidokas practicing other styles. Additionally, the participants had no continual training for about one year before the assessment session due to the COVID-19 pandemic. Although all the Aikidokas had at least one year of experience prior to the pandemic, this one-year suspension of training could have contaminated the results. Moreover, the evaluation of the elbow joint proprioception alone in the present study, would not provide useful information about the JPS of the adjacent joints; simultaneous evaluation of the adjacent joints should be considered in future studies. In addition, in the present study, proprioception was evaluated only passively through JPS. Further studies are warranted to measure active JPS as well as other sub-modalities of proprioception at the elbow joint of Aikidokas.

## Conclusions

Despite the repetitive stress applied to the elbow joint during the performance of Aikido techniques, the JPS of the elbow joint was not impaired in Aikidokas. It was also hypothesized that the lack of significant difference in isokinetic strength of elbow muscles between Aikidokas and healthy non-athletes, or the lack of an acceptable correlation between IPS and muscle strength in Aikidokas, might be attributed to the soft nature of Aikido.

## Data Availability

The datasets used and/or analyzed during the current study are available from the corresponding author on reasonable request.

## List of abbreviations

BMI: Body Mass Index
CE: Constant Error
CNS: Central Nervous System
E: Total Variability
JPS: Joint Position Sense
VE: Variable Error
